# Editorial: Public Health Nutrition: Assessing Evidence to Determine Policy and Practice

**DOI:** 10.3389/fpubh.2019.00021

**Published:** 2019-02-14

**Authors:** Alessandra Lafranconi, Sumantra Ray, Giuseppe Grosso

**Affiliations:** ^1^School of Medicine, Università degli Studi di Milano Bicocca, Milan, Italy; ^2^Department of International Health, Care and Public Health Research Institute (CAPHRI), Faculty of Health, Medicine and Life Sciences (FHML), Maastricht University, Maastricht, Netherlands; ^3^NNEdPro Global Centre for Nutrition and Health, Cambridge, United Kingdom

**Keywords:** food safety, food security, environmental factors, human nutrition and health, education tools, children, weight loss intervention, workplace health promotion

The concept of public health nutrition embraces many diverse areas, spanning from food safety, and food security, to environmental sustainability as well as human nutrition and health.

The present collection includes papers that touch upon all the aforementioned areas, providing a fascinating and variegated scenario of what public health nutrition stands for and how it may at times be overlooked in the twenty-first century.

With regards to **food safety**, Prosperini et al. present a review on a mycotoxin, Enniatin, and its specific profile of toxicology. The need of such review lies at the interface between science and policy, and attempts to respond to a specific mandate, whereby the European Commission asked the European Food Safety Authority (EFSA) for a scientific opinion, to quantify the risks related to acute and chronic exposure to Enniatin. Whilst the answer was unambiguous for acute toxicity (i.e., such toxins do not represent concerns for human health), there were no conclusions about chronic toxicity, mainly because of the complexity of performing a complete risk assessment for dietary exposure to the toxin itself. Moreover, the authors touch on some recent findings on carcinogenesis: Enniatin has been found to have anti-cancer actions, both *in vitro* and *in vivo*, at multiple cellular levels.

**Food security issues**, which are often dealt with at the national level, have different distributions in developing and developed countries (1); nevertheless, food insecurity constantly affects the most vulnerable classes of any given population, typically the children and the elderly. Gurung et al. focus on children affected by tuberculosis (TB) in Nepal, thus analyzing the conditions of an extremely vulnerable population segment. The authors performed a cross-sectional descriptive study to assess the dietary intake and nutritional status of children affected by TB, and found that about one-fifth of them did not consume a sufficient amount of calories, compared to the recommended daily average. Acting in a vicious circle, undernutrition increases the risk of TB progression into active disease, thus leading to further weight loss. Such data call for dietary assessments and nutritional treatments in children affected by TB.

According to the concept of planetary health (2), food security and **environmental factors**, such as greenhouse gas emissions (GHGEs), water depletion, land use, food waste, and ecosystem exploitation are highly entangled (3). Lacour et al. carried out a life-cycle assessment (LCA) study to compare GHGEs, energy demand and land occupation in a plant-based diet versus an animal-based diet, using a French cohort as baseline for the simulation. They found that those diets with a higher pro-vegetarian score (i.e., those diets with a preference for plant-based food) were significantly associated with lower GHGEs, lower energy demand and lower land occupation. Moreover, they investigated the modulation of such relationship, between type of diet and environmental impacts, by organic consumption; the authors found that organic food consumption modified the association between pro-vegetarian score and environmental impacts for plant-based diets, but not for animal-based diets.

Last, but not least, we included a number of papers on **human nutrition and health**. Nutrition has received significant attention in the last few years due to the increase in research done on its impact on non-communicable chronic and degenerative diseases. **Education tools** for healthcare professionals and for the general public are highly requested, as described by Crowley et al. massive open online courses might meet such needs, to counteract bad science and to provide practical help, especially in countries with low resources.

Progress in global, national, and local nutrition policies has been made, for instance with regards to specific population subgroups such as children and workers, where interventions to change the current food environment and, consequently, food habits, have been proposed.

Orlando et al. reviewed the link between obesity and hypertension in **children**, highlighting the role of fructose intake and the consequent rise in uric acid, which is a well-known independent risk factor for hypertension in children and adults. The authors then focused on nutritional policies aimed at decreasing the intake of fructose through actions on sweet drinks and carbonate beverages; effective actions appeared to be mainly carried out in school settings. Nevertheless, no data on cost-effectiveness of such policies have been presented.

Similarly, **workplace health promotion interventions** have been deemed effective. Korre et al. sketched their findings from their previous research on firefighters (a population at high risk of cardiovascular diseases, and often characterized by poor diet) and proposed a prospective intervention to modify their food environment through the adoption of Mediterranean diet, which has been shown to be palatable to them.

Another prospective intervention has been undertaken by Golubic et al. severely obese patients have been enrolled in a **multidisciplinary weight loss intervention**, prior to bariatric surgery. Interestingly, changes in weight were not-significantly modified by smoking or employment status: the intervention seemed to work homogeneously, despite the presence of social inequalities among the participants.

Taken together, the papers of our collection show research gaps in the following areas: measurement of actual costs of interventions, projection of impacts derived from nutrition policy implementations, and cost-effectiveness analysis of such interventions.

It is therefore unclear which evidence could be used to develop programmes and policy interventions that aim to improve health. This depends upon the different perspectives of researchers and decision-makers: the former, who are focused on knowledge generation, and the latter who are focused on assessing the disease burden that could be prevented or reduced by an intervention, and how feasible those interventions might be.

Especially in the European context, where a multitude of different legislation impacts our food environment and nutritional settings produce a variable science and policy lab, it is highly desirable to reach a consensus on minimum requirements that should apply to scientific evidence on nutrition interventions and policies. Only a solid assessment of costs and expected impacts can guide the design and implementation of a public health nutrition agenda which represents an interplay between heath and economic outcomes determined by food availability, dietary choices, and nutritional status of the population as well as how this can be modulated at each stage through effective risk assessment as well as risk management.

## Author Contributions

This Editorial has been written by AL, SR, and GG in order to summarize the articles (with their main findings) included in the Research Topic. All authors contributed to the Editorial, and they agree to the submitted version.

### Conflict of Interest Statement

The authors declare that the research was conducted in the absence of any commercial or financial relationships that could be construed as a potential conflict of interest.
